# Comparing the trueness of seven intraoral scanners and a physical impression on dentate human maxilla by a novel method

**DOI:** 10.1186/s12903-020-01090-x

**Published:** 2020-04-07

**Authors:** Zsolt Nagy, Botond Simon, Anthony Mennito, Zachary Evans, Walter Renne, János Vág

**Affiliations:** 1 Department of Conservative Dentistry, Semmelweis University, Szentkirályi utca 47, Budapest, H-1088 Hungary; 2 Department of Oral Rehabilitation, Medical University of South Carolina College of Dental Medicine, Charleston, SC USA; 3 Department of Stomatology, Medical University of South Carolina College of Dental Medicine, Charleston, SC USA

**Keywords:** Digital impression, Intraoral scan, Scanning origin, Physical impression, Trueness, Human cadaver

## Abstract

**Backgrounds:**

Intraoral scanner (IOS) accuracy is commonly evaluated using full-arch surface comparison, which fails to take into consideration the starting position of the scanning (scan origin). Previously a novel method was developed, which takes into account the scan origin and calculates the deviation of predefined identical points between references and test models. This method may reveal the error caused by stitching individual images during intraoral scan. This study aimed to validate the novel method by comparing the trueness of seven IOSs (Element 1, Element 2, Emerald, Omnicam, Planscan, Trios 3, CS 3600) to a physical impression digitized by laboratory scanner which lacks linear stitching problems.

**Methods:**

Digital test models of a dentate human cadaver maxilla were made by IOSs and by laboratory scanner after polyvinylsiloxane impression. All scans started on the occlusal surface of the tooth #15 (universal notation, scan origin) and finished at tooth #2. The reference model and test models were superimposed at the scan origin in GOM Inspect software. Deviations were measured between identical points on three different axes, and the complex 3D deviation was calculated. The effect of scanners, tooth, and axis was statistically analyzed by the generalized linear mixed model.

**Results:**

The deviation gradually increased as the distance from scan origin increased for the IOSs but not for the physical impression. The highest deviation occurred mostly at the apico-coronal axis for the IOSs. The mean deviation of the physical impression (53 ± 2 μm) was not significantly different from the Trios 3 (156 ± 8 μm) and CS 3600 (365 ± 29 μm), but it was significantly lower than the values of Element 1 (531 ± 26 μm), Element 2 (246 ± 11 μm), Emerald (317 ± 13 μm), Omnicam (174 ± 11 μm), Planscan (903 ± 49 μm).

**Conclusions:**

The physical impression was superior compared to the IOSs on dentate full-arch of human cadaver. The novel method could reveal the stitching error of IOSs, which may partly be caused by the difficulties in depth measurement.

## Background

An increasing number of dentists are purchasing and implementing intraoral scanners (IOS) for making optical impressions of oral soft and hard tissues as an alternative to traditional impressions, which are generally poorly tolerated by patients [1–3]. The area of application previously consisted mostly of quadrant imaging [4–8]. However, as systems become faster, more accurate, and easier to use [9, 10], more clinicians are scanning complete arches and wide-spanning dental restorations [11–13].

A considerable number of IOSs are available on the dental market, and this number is growing each year [14]. The characteristic feature between imaging processes in IOS is that their field of view is unable to capture all surfaces simultaneously [15, 16]. When the desktop scanners in dental laboratories scan the full arch, the first image covers the entire object from a single sensor position, especially scanners with structured light, therefore generating an overall mesh even if some details are missing in hidden areas [17, 18]. The missing information is acquired from a different perspective during the rotation of the object, and the following images are registered into the original mesh employing the multiple overlapping regions [19, 20]. Contrastingly, small overlapping images are taken by IOS during linear movement of the device on the arch and are later merged with a software algorithm called “stitching” [21–23]. With the linear progression of imaging the dental arch, there are more and more surfaces to be integrated, eventually resulting in some degree of inaccuracy in the digital model [16, 24]. Accuracy, as defined by the International Organization for Standardization (ISO) 5725, consists of both trueness and precision [25]. Trueness refers to the closeness of agreement between the arithmetic mean of a large number of test results and the true or accepted reference value. Precision refers to the closeness of agreement between test results. Accordingly, the trueness of the intraoral scan often gradually decreases as the scan progresses away from the point of scan origin [21–23, 26]. In order to reveal the trueness deviation due to the stitching, a new methodology was developed [27] in which superimposition was performed at the scanning origin only after carefully aligning this initial data point. Contrastingly, the most common method for trueness testing is the superimposition of the whole scans onto the reference standard file in a way to align the entire model with no consideration for scanning origin [4, 28]. Previously it was also demonstrated that if identical points were marked on the scanned dental arch (2 points per tooth, on both the master and test model), a higher deviation could be measured than by the mean deviation of the whole arch surface [27]. Traditionally a study model is aligned to the reference standard model using an iterative best-fit algorithm. This forces the two objects to align as much as they can, independent of the fact that in most cases, these two pixels are not identical in an anatomical or true position. In the second step, it calculates the cumulated average deviation based on this alignment, which results in an artificially low average deviation [9]. Therefore, using this traditional method of trueness evaluation results in the study model appearing more true than it actually is. To address this limitation, Vag et al. described a comparison method that had a higher sensitivity and was able to detect trueness deviations in scan patterns when the full-arch surface deviation method could not [27]. The novel method consists of two distinctive features contrary to the full-arch surface comparison method. First, multiple points are defined on the reference model, two for each tooth on the occlusal surface. Each of the two points is copied to the test model after local best fit alignment at the respective tooth. In this way, identical points can be selected on the two models avoiding any manual error in trying to select the same spot on different models. Second, test and reference models are re-aligned at the tooth where the scan was started (scan origin) and the deviation between the identical points is calculated at each tooth.

Recently the surface comparison method found only minimal statistical differences between seven IOSs with different optical principles and supported by different software types [13]. This study was conducted on a human cadaver to simulate better the actual optical properties of the human dentate arch. The hypothesis is that with this novel method [27] it is possible to detect more specific differences in trueness between scanners. The raw data files from the previous study were readily available, and therefore, in this study, we aimed to calculate the trueness by the identical point method on data that had previously been analyzed by the full arch surface comparison method [13]. The original raw data also included a digitized form of a physical impression. This inclusion allows for a further hypothesis to be answered. The increased deviation measured with the origin merging method may be just a consequence of less accurate (due to the smaller surface) model merging. In other words, it might be possible that the measured error is due to the inferior model merging of the test and reference models rather than true distortion. If the digitization of a physical impression did not show any accumulated distortion after local best fit at a theoretical scan origin, then the role of the weaker alignment in generating a distortion artifact could be excluded as a variable.

The primary aim of this study was to compare the distortion proceeding across the teeth of intraoral scans to the digitized physical impression. The secondary objective was to measure deviation for each axis separately in order to find the axis that is influenced the most by stitching errors during complete arch scanning. The third aim was to compare the trueness of seven IOSs by measuring the overall mean deviation from the reference standard scan.

## Methods

**Fig. 1:**
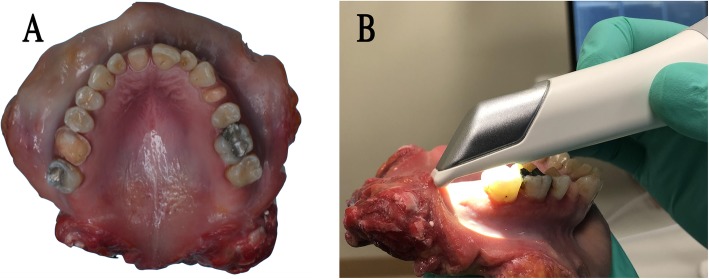
**a** Cadaver maxilla with teeth (tooth #3, #8, #12) prepared for a crown, **b** Scanning initiated at the scanning origin (tooth #15)

For modeling the effect of various optical properties of the substrate, a maxilla from a fresh human cadaver with a complete dentition was examined, during which three teeth were prepared for full tooth coverage crowns, and some teeth contained amalgam or composite fillings (Fig. 1a) [29, 30]. The dissected maxilla was kept on 4 °C and wet during the test in order to conserve the condition of tissues. The use of cadaver tissue in this study was deemed exempt from review by the Institutional Review Board for Human Research in Medical University of South Carolina (Pro 77,251). According to this, no informed consent was necessary to use cadaver tissue.

The human dissected maxilla was digitized with an industrial 3D scanner (ATOS III Triple Scan 3D optical scanner, GOM, Braunschweig, Germany, fringe pattern projection, and three-way triangulation), and was the basis for comparison with the intraoral scans. Seven different intraoral scanners were selected for evaluation. Inbuilt technology by scanners was the following, Trios 3 (3Shape, Copenhagen, Denmark, LED structured light confocal microscopy, software v1.17.2.4.), CEREC Omnicam v2.24 (Dentsply Sirona, York, PS, US, white structured light, optical triangulation, and confocal microscopy, software v4.5.2.), CS 3600 (Carestream, Dental Atlanta, GA, US, 4 LED, blue, green, red, UV structured light, active speed 3D video, software 3.1.0), iTero Element 1 and Element 2 (Align Technologies, San Jose, CA, US, red laser parallel confocal imaging, v1.5.0.361), Planmeca Emerald (Planmeca, Helsinki, Finland, three-color laser projected pattern, triangulation, software v5.9.4), Planscan (Planmeca, Helsinki, Finland, laser projected pattern, triangulation, software v5.9.4) [28]. All scans were performed five times by a single operator who was experienced in the specific system and the manufacturers recommended scan pattern was used for each individual scanner. These scans included all teeth in the maxilla, the approximal parts of prepared areas, gingival regions and the palate. All scanning started at tooth #15 (universal notation) (Fig. 1b). Five traditional polyvinylsiloxane (PVS) impressions (V-Posil, Voco GmbH, Germany) were also made using a two-phase simultaneous technique with stock trays, and a precision stone model (Silky Rock, Whip Mix Corp., Louisville, KY, US) was poured from each impression. All stone models were digitized with a dental desktop laboratory scanner (D800, 3Shape) using the highest possible accuracy (50 μm) in order to imitate the usual workflow between the dentist and the dental technician. The environmental conditions were kept constant, and the specimen was moistened and kept on ice after each scan.

**Fig. 2:**
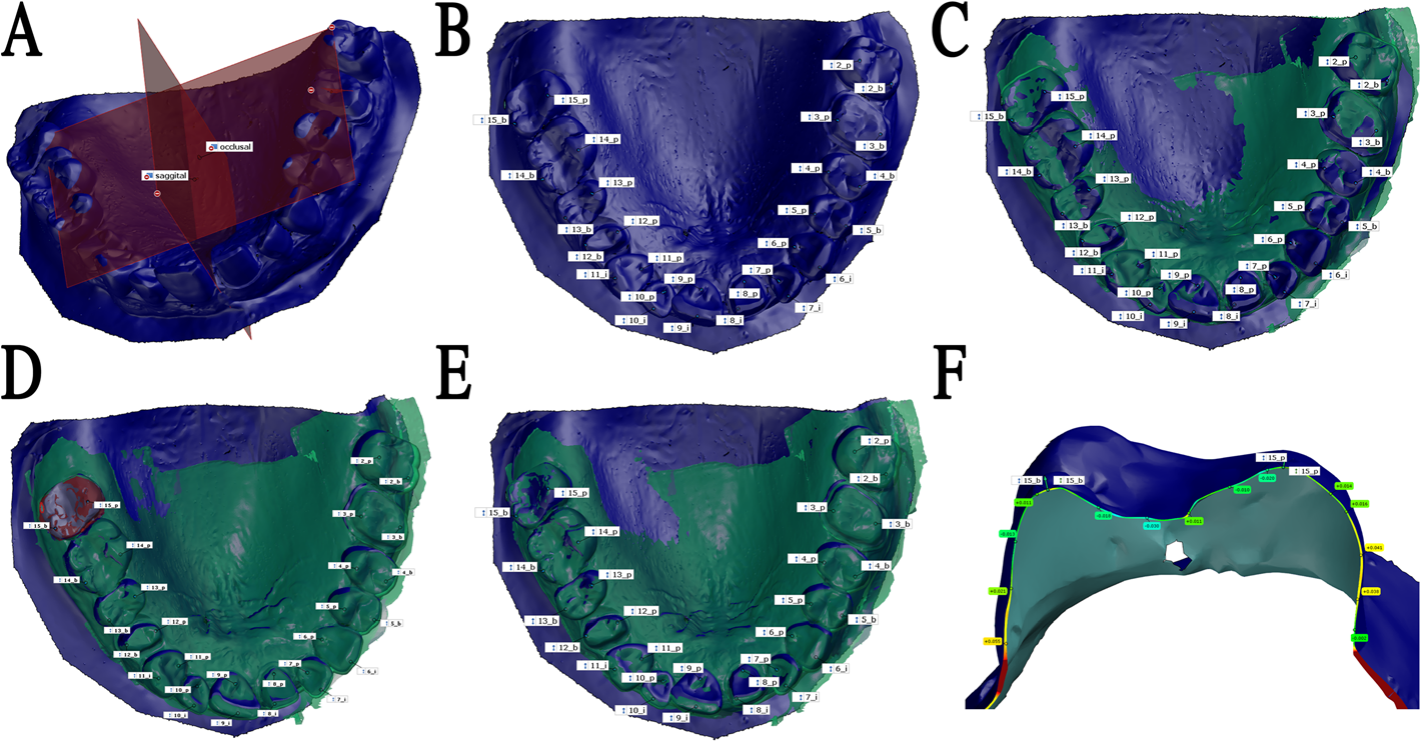
A representative example showing the method of superimposition of a test scan made by Planscan (green) to the reference scan (blue) (**a**) Construction of a local coordinate system by fitting dental planes on the references scan in GOM Inspect, (**b**) Selecting two points for each tooth on the reference model, (**c**) Prealignment of the test scan to the whole arch, (**d**) Selection of tooth 15 (red surface, scanning origin) for local best fit, (**e**) Alignment using local best fit at tooth 15, (**f**) Copy points of 15_b and 15_p from reference model to the test model. Labels with color background indicate surface distances between the two models along the section line

All test scan files were exported in standard tessellation language (STL) format from the dedicated software of each system. The STL files were imported to the GOM Inspect software (GOM GmbH, Germany) for the comparative tests according to the novel, identical point method described previously [27]. First, a proper coordinate system was established on the master model (reference standard), where axes X, Y, and Z represented the occlusal and sagittal planes (Fig. 2a). After this, two measurement points were selected on each tooth at specified locations on the model surface, which were used as reference points (28 points total) (Fig. 2b). Intraoral scans were evaluated individually, during which an automatic surface-based superimposition with the master model was performed in the first step by the software (Fig. 2c). In the next step, each tooth was outlined (Fig. 2d) to determine and correlate the selected reference points using the local best-fit algorithm on each tooth one by one (Fig. 2e). When the two models aligned at a specific tooth, the respecting two points were copied from the master to the test model (Fig. 2f). Finally, the fitting of the two samples (master and intraoral scan) was reset to the origin point of the scans, at tooth #15. The deviation values between identical points were registered along all three axes of the proper coordinate system. The mean complex deviation in 3D was calculated by Pythagoras theorem from the 3 axis vectors.

### Outcome variables

- The complex 3D deviation was measured for each tooth in an order related to the scanning direction through the whole arch. It may represent the accumulated deviation due to the stitching of the consecutive images.
- The deviation for each axis was statistically compared for each scanner separately to reveal the axis having the highest deviation. The scans were always started on the occlusal surface, and this view was assigned to the z-axis. Therefore, the deviation measured in the z-axis indicates the error in depth measurement, which is the unique feature of IOS distinguishing from the 2D camera.
- The complex 3D deviations were averaged across the teeth, and these values were compared between IOSs and between IOSs and physical impression. This mean value indicates the overall trueness of the scanners, and its standard deviation represents the precision (i.e., reproducibility) [31–33].

### Statistics

The data were exported to MS Excel for rearrangement and then exported for further statistical analysis into SPSS 25 (IBM SPSS Statistics for Windows, Version 24.0. Armonk, NY:IBM Corp). Data in the text and the figures are presented as mean ± standard error of the mean, except Figs. 6 and 7 where the mean ± standard deviation (SD) is shown. Deviation values were analyzed by the generalized linear mixed model with gamma distribution and log-link function using restricted maximum likelihood estimation. In the first model, the complex deviation values (the combination of the absolute values of the three vectors, x, y, z) were analyzed with two main factors, tooth and scanner, and their interaction. In the second model, the deviation measured on the three-axis separately was analyzed, including the main effect of scanner and axis and scanner*axis interaction. The *p* values were adjusted using the Sidak method for pair-wise comparison with an alpha value set at 0.05. The standard deviation of the complex deviation averaged across the tooth was not evaluated by means of statistics as no direct superimposition was made between test scans.

## Results

### Complex 3D deviation by tooth

**Fig. 3:**
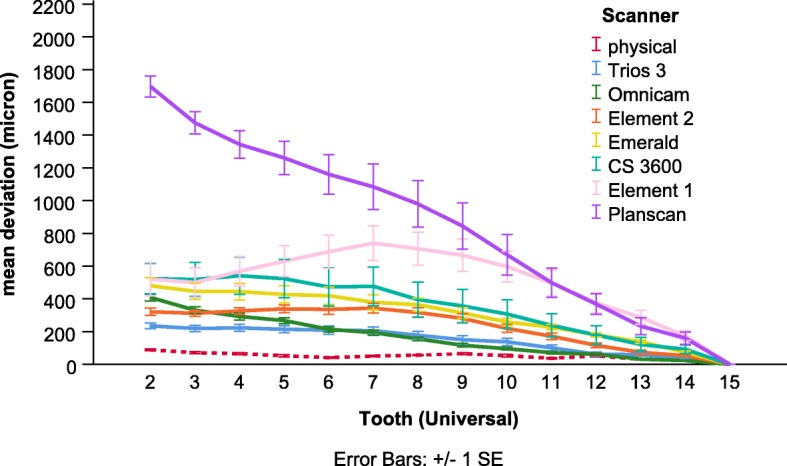
Comparison of the mean complex deviation measured in 3D between scanners as a function of the tooth number

**Fig. 4:**
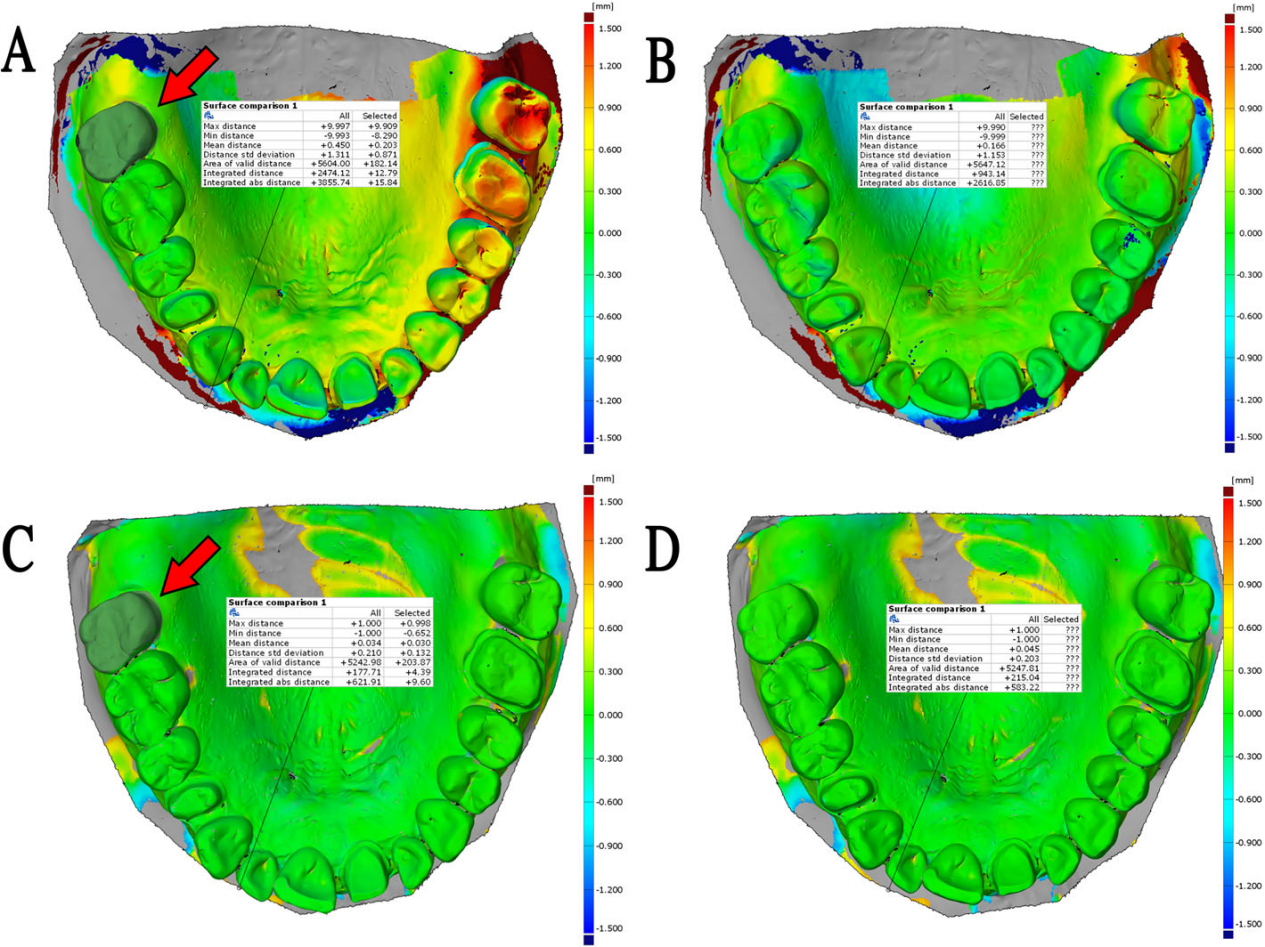
Full arch surface comparison of two selected scans from Planscan (**a**, **b**) or Physical impression (**c**, **d**) group. **a** In the case of local best fit at scanning origin (red arrow, tooth 15), the surface deviation on the tooth 15 was 87 μm, where on the whole arch, it was 688 μm. **b** In the case of best fit on the full arch (conventional method), the surface deviation on the whole arch was 463 μm. **c** In the case of local best fit at scanning origin (red arrow, tooth 15), the surface deviation on tooth 15 was 47 μm, where on the whole arch, it was 119 μm. **d** In the case of best fit on the full arch (conventional method), the surface deviation on the whole arch was 111 μm. The scale of color bars set equally on each figure. Little difference can be seen between **b** and **d** figures contrary to the differences between **a** and **c**, indicating the importance of the scan origin

**Fig. 5:**
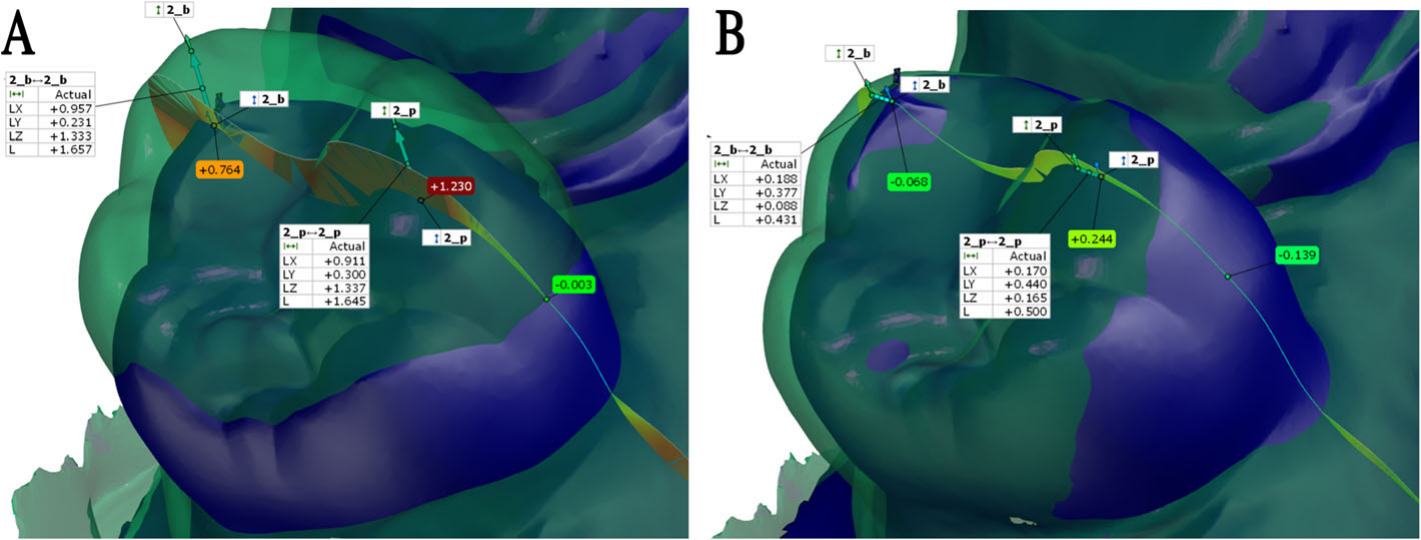
The cross-section through the two reference points of the terminal tooth (#2) in the case of local best fit at scanning origin (tooth 15) (**a**) and in the case of full arch alignment (**b**). The two double arrows (jade color) connects the two identical points between reference scan (blue) and test scan (transparent green, made by Planscan) in the 3D. The 3D distances (L) are shown in the attached tables. The colorful vectors in the cross-section, indicating the magnitude of the surface comparison values. Labels with color background show that surface deviation values at the points (2_b and 2_p) on the reference scan are considerably lower than the distances between identical points (L values). Furthermore, at the crossing point of the two surfaces (the label on the palatal site), it tends to zero. Multiple crossing points through the arch decrease the overall surface deviation value

In the first model, both the main effects, the tooth (*p* < 0.001) and scanner (p < 0.001), and their interaction (p < 0.001) were significant. The significant interaction effect is shown in Fig. 3. The value is constantly rising to start from scanning origin, and this tendency is present for almost all scanners but Element2 and Element1, where the trend of deviation turns and decreases at the anterior region. The most deviation occurred with Planscan. Representative surface comparison of a test scan made by Planscan shows only a little deviation in the case of full-arch alignment (Fig. 4a) contrary to the best fit alignment at the scan origin (Fig. 4b). The 3D image of the terminal tooth (#2) demonstrates the accumulated distortion measured by the deviation between the identical points at the cusps (Fig. 5), which is much higher than the deviation between surfaces. This increased potential of deviation from scan origin to termination cannot be seen in the physical model (Fig. 4c).

### Deviation by axis

**Fig. 6:**
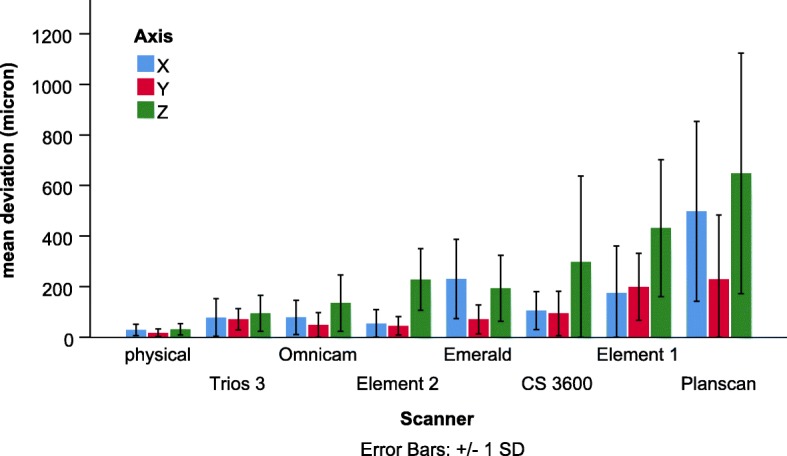
Comparison of the mean deviation of scanners across the whole arch between three axes

The scanner (*p* < 0.001), axis (p < 0.001), and their interaction (*p* < 0.01) were significant in the second model. Overall the mean deviation (full-arch) on the axis Z (122 ± 11 μm) was significantly higher than on the axis X (73 ± 7 μm, *p* < 0.05) and on the axis Y (52 ± 5 μm, p < 0.05). But the significant scanner * axis interaction indicates that there are some differences in axis dependent deviation among scanners (Fig. 6). For most of the scanners, the complete arch deviation was the highest on axis Z, and values were similar on the other two axes. The only exception is the scanners from Planmeca (PlanScan and Emerald), where values for axis X were similar to values for axis Z.

### The mean deviation

**Fig. 7:**
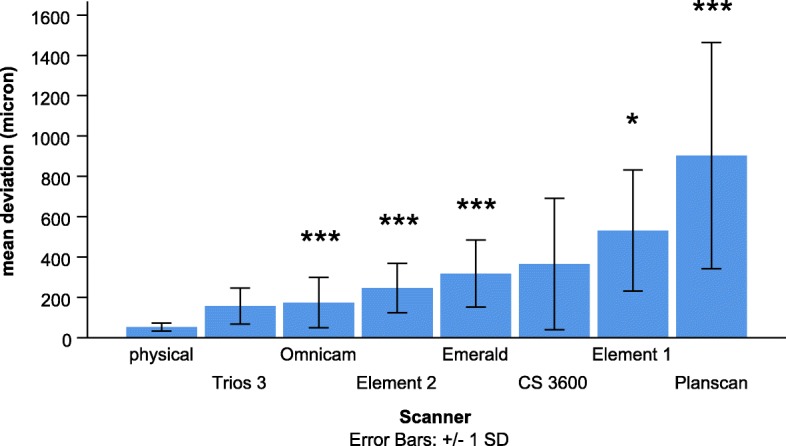
Comparison of the mean complex deviation between scanners averaged out for the whole arch. * indicates significant differences between physical impression and IOS, * *p* < 0.05, *** *p* < 0.001

In the first statistical model, the significant main effect of a scanner indicates differences between scanners in the complex deviation values averaged over the full arch (the overall trueness of the scanners) (Fig. 7). Pairwise post-hoc analysis showed that deviation of the physical impression (53 ± 2 μm) was significantly lower than the values of most scanners (Element 1, 531 ± 26 μm, *p* < 0.05; Element 2, 246 ± 11 μm, *p* < 0.001; Emerald 317 ± 13 μm, p < 0.001; Omnicam, 174 ± 11 μm, p < 0.001, Planscan, 903 ± 49 μm, p < 0.001), except the Trios 3 (156 ± 8 μm, *p* = 0.068) and CS 3600 (365 ± 29 μm, *p* = 0.208). The deviation by the Trios 3 was significantly lower than the Planscan (p < 0.001) and the Emerald (p < 0.05), and the Omnicam had a lower value than the Planscan (p < 0.001) and the Emerald (*p* < 0.01). The Element 2 and the Emerald had a significantly lower value than the Planscan (p < 0.01, p < 0.05, respectively). CS 3600 was not different from any other scanner. The deviation of Element 1 was not significantly different from other scanners.

The standard deviation in Fig. 7 represents the precision (i.e., reproducibility). Precisions were the following; physical impression (20 μm), Trios 3 (89 μm), Element 2 (123 μm), Omnicam (125 μm), Emerald (166 μm), Element 1 (300 μm), CS 3600 (326 μm), Planscan (561 μm). This order is very similar to the order of trueness values.

## Discussion

Recent studies have demonstrated comparable trueness of direct IOS digitization of full-arches to indirect digitization with the conventional impression being poured and digitized by a laboratory scanner. In vitro, a polymeric full-arch model was replicated by either indirect method using polyether impression material or by True Definition scanner (TD) [34]. The 3D linear shift between the two endpoints of the scan (between second molars) was 122 μm for TD scanner, and it was 174 μm for the indirect method. Using a similar approach for evaluation the trueness [12] the 3D linear shift between the two endpoints of the scan was 287 μm for iTero Element scanner (the version was not given), and it was 257 μm for the indirect method in vitro, but in vivo, the intraoral scan resulted in better trueness (305 μm) than the indirect method (517 μm). The method used in these papers resembles our identical point method in terms of that it measures the deviation of predefined points at the most distant region; that is why the values for the iTero Element were comparable to our result (246 μm). In another study [6], the trueness of indirect digitization of the full arch model was compared to seven IOS. The indirect digitization method resulted in significantly better trueness (16 μm) than any other IOSs; 49 μm for Trios 3, 58 μm for Carestream 3600, 89 μm for Medit i500, 63 μm for iTero Element 2, 48–90 μm for Omnicam models. This data was considerably lower than our results, probably due to the traditional surface comparison method. Similar results were found in another study [35]. A new method was developed to measure full-arch trueness in patients by bonding four spheres onto the teeth (around second molars and first premolars) surface with known, constant distances. After intraoral scan or indirect digitization, the local best fit was applied on the spheres and the surface deviation was calculated. The indirect method yielded the best trueness (15 μm) followed by TD (23 μm), Trios (37 μm), CEREC Omnicam (214 μm).

Our result employed in a dentate human cadaver arch showed no increase in deviation as a function of distance for physical impressions, which is consistent with a previous study [24]. The authors found little increase (from 15 μm to 67 μm) in deviation from the theoretical scan origin (left second upper molar) to the end of scanning (contralateral molar) for physical impression, but a more substantial increase was observed for IOS. The values ranged from 15 μm to 205 μm for the CS 3600 (with a mean of 119 μm) and from 10 μm to 227 μm for the Trios 3 (with a mean of 184 μm). They were somewhat different from the present results (365 μm and 156 μm respectively) for this specific scanner, but we used cadaver instead of a stone model, and the identical point method was applied instead of the surface comparison method which makes a considerable difference (Fig. 7.). Similarly to the present study, the trueness averaged across the full arch for Planscan found to be 661 μm measured by the identical point method with the consideration of scanning origin, and this was three times more than with the full arch surface comparison (197 μm) [27]. Thus, the difference between the above study and the present one could be explained by the differences between method, i.e., the surface versus identical point deviation.

If the alignment of the actual scan to the reference model is done at the tooth of scanning origin, then the deviation grows as distance increases from the origin [24, 27]. The surface alignment at one tooth involves fewer pixels; therefore, it could be speculated that the local best-fit algorithm limited to one tooth has less accuracy compared to full arch alignment. It may cause an artifact of a constant linear increase in deviation from the starting point. However, the increase was neither linear nor constant. In this study, a physical impression captured by a laboratory scanner was also analyzed by fixing the experimental model to the control model at what would be the scan origin during alignment. The present result confirms that accumulated deviation is not an artifact but instead is caused by the stitching mechanism, which is specially related to IOS, but not to the laboratory scanners [16, 36]. This problem is likely the main reason that physical impressions continue to have higher trueness for full arches versus direct intraoral scanning [6, 35, 37, 38]. The tooth wise kinetics of the deviation suggests that the actual deviation at a specific tooth could be much more than the average full arch deviation. Thus initiation of scanning should be done as close to the restoration site as possible to minimize model distortion.

The novel method could also perform the measurements separately along three different axes according to the dental planes on the arch (occlusal, sagittal, and transversal). Scans were started on the occlusal, resulting in axis Z showing the distance between the occlusal surface and the scanner. The investigated IOSs use different optical mechanisms in order to determine depth information [39–41]. The deviation measured along the z-axis can indicate the differences between various types of hardware. This sets an internal coordinate system for stitching further images together. The hardest step is defining the depth parameter, which differentiates a 3D scanner from a 2D camera. Similarly to another study [34], we found that axis Z is the most inaccurate dimension.

Previously, the raw data used in this study was analyzed by the surface comparison method and revealed no statistical difference among scanners in terms of trueness and precision for full-arch imaging with the exception of the Planscan, which showed significantly higher deviation [13]. The authors concluded that it might require a higher sample size to reveal statistical differences. Our novel method using identical points with alignment at the scanning origin on the compared objects was capable of distinguishing between the trueness of scanners investigated using the same raw data. That is similar to a previous study showing this novel method illustrated a significant effect of the scanning patterns on trueness when compared to the commonly used traditional best-fit surface comparison method [27]. This finding confirms the increased sensitivity and statistical power of the new method (Fig. 6.). The higher specificity could prove increasingly valuable as the newer generation of IOS is more and more accurate [4, 6]. In this study, newer versions from the same manufacturer produced better trueness (Element 1 vs. Element 2 and Planscan vs. Emerald) similarly to other studies [6, 24]. Apart from hardware research, manufacturers make significant investments in performing software R&D to continually improve the accuracy of IOS (both trueness and precision), which can also be experienced by users after a version update [27, 42]. Furthermore, many factors can influence accuracies such as scanning pattern [5, 43, 44] or substrate reflectance [29, 30] software settings and upgraded versions [6, 42]. Accuracy studies must be reevaluated from time to time due to this constant development.

According to our results and others [6, 13, 28], it is not surprising that precision and trueness had a positive correlation. The precision is statically equal to the standard deviation of the trueness; therefore, a non-significant difference in trueness between scanners does not necessarily mean that one scanner is better than the other. High standard deviation (low precision) could mask the differences in trueness values. In this study, the best example is that the difference between CS 3600 and the physical impression was not significant due to the high standard deviance (low precision) of the CS 3600, but we cannot conclude that this is a perfect scanner. Precision also has a high clinical relevance because a restoration needs to fit in every case regardless of which scan, usually only one, is it created from.

Determining the precision of the IOS from in vivo clinical assessment is a fairly simple method, as the 3D models generated through the repeated, consecutive scanning of the dental arch can easily be compared with appropriate analytical software that can superimpose these models with each other [6, 12, 13, 24, 44–46]. However, direct evaluation of the trueness value is not possible in vivo, as the reference model can only be created using indirect extraoral methods with physical or optical tracking devices. A dental arch constrained within a human’s head cannot be transferred into these devices. Therefore, trueness values are usually assessed in vitro, on the manufactured physical samples [6, 24, 44–46]. Recently, it has been demonstrated that the optical properties of various materials influence the accuracy of IOS, which is likely also true of mucosal tissues and other restorative and optical impression material combinations [29, 30]. Therefore, this study represents a further step toward a more clinically relevant analysis by using a cadaver with representative tissues compared to artificial materials and substrates.

However, other clinical conditions were not simulated in our study. The substantial differences in the deviation between intraoral scan and extraoral scanning using the same protocol were explained by the patient movement, limited intraoral space, intraoral humidity, and saliva flow in another study [36]. The cadaver tissue in our study was removed from the body make the scanning easier for the operator, and it was free of saliva, but it kept wet by water. In a study [47], where artificial saliva was applied on the model surface, the intraoral scans were distorted considerably compared to other in vitro studies [6, 24], probably due to the bubbles formed on the surface. Another limit of our method could be that although the mean surface deviation for the local best fit applied at a single tooth was very low (mean = 18 μm, standard deviation = 13 μm, *n* = 574), in some case (e.g., a Planscan case) it went up to 146 μm. This deviation could be considered the error in the automatic copy of the points from the reference scan to the test scan. However, this deviation is much less than the differences between the deviation measured on the surface using non-identical points (conventional method), and the deviation measured between identical points regardless of full arch alignment or alignment at scan origin was used. The manual selection of these points may also involve some uncertainty in the novel method. The points were selected on the cusps on the molars and the premolars and on the incisal edge on the anterior. These areas are special in terms of having high deflection. If we had selected some points on the smooth lateral surfaces on the tooth, we might have been closer deviation to the conventional surface comparison method. Contrary to a pre-designed geometrical object (i.e., an engineered object such as an implant scan body), the teeth are amorphous. Consequently, there are no special points on it. Another limitation that this method still suffers from the same problem as the conventional method, such as there is no physical limit of superimposition. The test scan could go in and out of the reference scan resulting in a negative (under the surface) and positive (above the surface) deviation values. In reality, it is not possible as the designed framework cannot sink into the virtual model (similarly to the physical stone model). Recently it was demonstrated [48] that this kind of virtual superimposition method may underestimate the more real physical alignment. This problem only partly eliminated by the alignment at the scan origin.

## Conclusions

Our novel technique seems suitable to reveal the stitching error, which may be one of the weakest points of IOSs. This feature may contribute to the increased power to find statistical differences in trueness between IOSs. It was also demonstrated that the highest deviation of IOSs mostly occurs in depth measurement. In human cadaver, the dentate full-arch physical impression has the best trueness and precision. However, the relatively newer IOS systems (Trios 3, Omnicam, Element 2, Emerald) have clinically acceptable results as well.

## Data Availability

The datasets used and/or analyzed during the current study are available from the corresponding author upon reasonable request.

## Abbreviations

IOS: Intraoral scanner
PVS: Polyvinylsiloxane
STL: Standard tessellation language
TD: True definition scanner

## References

[1] Uhm SH, Kim JH, Jiang HB, Woo CW, Chang M, Kim KN (2017). Evaluation of the accuracy and precision of four intraoral scanners with 70% reduced inlay and four-unit bridge models of international standard. Dent Mater J.

[2] Marghalani A, Weber HP, Finkelman M, Kudara Y, El Rafie K, Papaspyridakos P (2018). Digital versus conventional implant impressions for partially edentulous arches: an evaluation of accuracy. J Prosthet Dent.

[3] Tomita Y, Uechi J, Konno M, Sasamoto S, Iijima M, Mizoguchi I (2018). Accuracy of digital models generated by conventional impression/plaster-model methods and intraoral scanning. Dent Mater J.

[4] Renne W, Ludlow M, Fryml J, Schurch Z, Mennito A, Kessler R (2017). Evaluation of the accuracy of 7 digital scanners: an in vitro analysis based on 3-dimensional comparisons. J Prosthet Dent.

[5] Mennito AS, Evans ZP, Lauer AW, Patel RB, Ludlow ME, Renne WG (2018). Evaluation of the effect scan pattern has on the trueness and precision of six intraoral digital impression systems. J Esthet Restor Dent.

[6] Ender A, Zimmermann M, Mehl A (2019). Accuracy of complete- and partial-arch impressions of actual intraoral scanning systems in vitro. Int J Comput Dent.

[7] Davidovich E, Dagon S, Tamari I, Etinger M, Mijiritsky E. An Innovative Treatment Approach Using Digital Workflow and CAD-CAM Part 2: The Restoration of Molar Incisor Hypomineralization in Children. Int J Environ Res Public Health. 2020;17(5):1499. 10.3390/ijerph17051499 PMC7084897 32110963

[8] Davidovich E, Shay B, Nuni E, Mijiritsky E. An Innovative Treatment Approach Using Digital Workflow and CAD-CAM Part 1: The Restoration of Endodontically Treated Molars in Children. Int J Environ Res Public Health. 2020;17(4):1364. 10.3390/ijerph17041364 PMC7068261 32093253

[9] Mangano F, Gandolfi A, Luongo G, Logozzo S (2017). Intraoral scanners in dentistry: a review of the current literature. BMC Oral Health..

[10] Aswani K, Wankhade S, Khalikar A, Deogade S (2020). Accuracy of an intraoral digital impression: A review. J Indian Prosthodont Soc.

[11] Kim RJ, Park JM, Shim JS (2018). Accuracy of 9 intraoral scanners for complete-arch image acquisition: A qualitative and quantitative evaluation. J Prosthet Dent.

[12] Keul C, Guth JF. Accuracy of full-arch digital impressions: an in vitro and in vivo comparison. Clin Oral Investig. 2020;24(2):735–45. 10.1007/s00784-019-02965-2 31134345

[13] Mennito AS, Evans ZP, Nash J, Bocklet C, Lauer Kelly A, Bacro T (2019). Evaluation of the trueness and precision of complete arch digital impressions on a human maxilla using seven different intraoral digital impression systems and a laboratory scanner. J Esthet Restor Dent.

[14] Al-Hassiny HA-H, A. (2019). Review of the intraoral scanners at IDS 2019.

[15] Goracci C, Franchi L, Vichi A, Ferrari M (2016). Accuracy, reliability, and efficiency of intraoral scanners for full-arch impressions: a systematic review of the clinical evidence. Eur J Orthod.

[16] Abduo J, Elseyoufi M (2018). Accuracy of intraoral scanners: A systematic review of influencing factors. Eur J Prosthodont Restor Dent.

[17] Claus D, Radeke J, Zint M, Vogel AB, Satravaha Y, Kilic F (2018). Generation of 3D digital models of the dental arches using optical scanning techniques. Semin Orthod.

[18] Ramiro GP, Hassan B, Navarro AF, Coronel CA, Cortes ARG, Baptista OHP, et al. Digitalization in Restorative Dentistry. Digit Restor Dent. 2019:7–39.

[19] Fukazawa S, Odaira C, Kondo H (2017). Investigation of accuracy and reproducibility of abutment position by intraoral scanners. J Prosthodont Res.

[20] Park J-M, Shim J (2019). Optical impression in restorative dentistry.

[21] Fisher B, McDonagh S, editors. Simultaneous registration of multi-view range images with adaptive kernel density estimation. Proceedings of the IMA 14th Mathematics of Surfaces. Birmingham: Institute of Mathematics and its Applications. 2013:31–62.

[22] Mao Z, Park K, Lee K, Li X (2014). Robust surface reconstruction of teeth from raw pointsets. Int J Numer Method Biomed Eng.

[23] Park S, Kang HC, Lee J, Shin J, Shin YG (2015). An enhanced method for registration of dental surfaces partially scanned by a 3D dental laser scanning. Comput Methods Prog Biomed.

[24] Park GH, Son K, Lee KB (2019). Feasibility of using an intraoral scanner for a complete-arch digital scan. J Prosthet Dent.

[25] Accuracy (trueness and precision) of measurement methods and results Part 1: General principles and definitions (ISO 5725e1:1994)1994.

[26] Rusinkiewicz S, Hall-Holt O, Levoy M (2002). Real-time 3D model acquisition. ACM Trans Graph.

[27] Vag J, Nagy Z, Simon B, Mikolicz A, Kover E, Mennito A (2019). A novel method for complex three-dimensional evaluation of intraoral scanner accuracy. Int J Comput Dent.

[28] Mangano FG, Hauschild U, Veronesi G, Imburgia M, Mangano C, Admakin O (2019). Trueness and precision of 5 intraoral scanners in the impressions of single and multiple implants: a comparative in vitro study. BMC Oral Health.

[29] Dutton E, Ludlow M, Mennito A, Kelly A, Evans Z, Culp A, et al. The effect different substrates have on the trueness and precision of eight different intraoral scanners. J Esthet Restor Dent. 2020;32(2):204–18. 10.1111/jerd.12528 31568660

[30] Bocklet C, Renne W, Mennito A, Bacro T, Latham J, Evans Z (2019). Effect of scan substrates on accuracy of 7 intraoral digital impression systems using human maxilla model. Orthod Craniofac Res.

[31] Di Fiore A, Meneghello R, Graiff L, Savio G, Vigolo P, Monaco C (2019). Full arch digital scanning systems performances for implant-supported fixed dental prostheses: a comparative study of 8 intraoral scanners. J Prosthodont Res..

[32] Guth JF, Keul C, Stimmelmayr M, Beuer F, Edelhoff D (2013). Accuracy of digital models obtained by direct and indirect data capturing. Clin Oral Investig.

[33] Shimizu S, Shinya A, Kuroda S, Gomi H (2017). The accuracy of the CAD system using intraoral and extraoral scanners for designing of fixed dental prostheses. Dent Mater J.

[34] Guth JF, Edelhoff D, Schweiger J, Keul C (2016). A new method for the evaluation of the accuracy of full-arch digital impressions in vitro. Clin Oral Investig.

[35] Kuhr F, Schmidt A, Rehmann P, Wostmann B (2016). A new method for assessing the accuracy of full arch impressions in patients. J Dent.

[36] Flügge TV, Schlager S, Nelson K, Nahles S, Metzger MC (2013). Precision of intraoral digital dental impressions with iTero and extraoral digitization with the iTero and a model scanner. Am J Orthod Dentofac Orthop.

[37] Ender A, Attin T, Mehl A (2016). In vivo precision of conventional and digital methods of obtaining complete-arch dental impressions. J Prosthet Dent.

[38] Malik J, Rodriguez J, Weisbloom M, Petridis H (2018). Comparison of accuracy between a conventional and two digital intraoral impression techniques. Int J Prosthodont.

[39] Syrek A, Reich G, Ranftl D, Klein C, Cerny B, Brodesser J (2010). Clinical evaluation of all-ceramic crowns fabricated from intraoral digital impressions based on the principle of active wavefront sampling. J Dent.

[40] Logozzo S, Zanetti EM, Franceschini G, Kilpelä A, Mäkynen A (2014). Recent advances in dental optics – part I: 3D intraoral scanners for restorative dentistry. Opt Lasers Eng.

[41] Richert R, Goujat A, Venet L, Viguie G, Viennot S, Robinson P (2017). Intraoral scanner technologies: A review to make a successful impression. J Healthc Eng.

[42] Haddadi Y, Bahrami G, Isidor F (2018). Effect of software version on the accuracy of an intraoral scanning device. Int J Prosthodont.

[43] Medina-Sotomayor P, Pascual MA, Camps AI (2018). Accuracy of four digital scanners according to scanning strategy in complete-arch impressions. PLoS One.

[44] Latham J, Ludlow M, Mennito A, Kelly A, Evans Z, Renne W. Effect of scan pattern on complete-arch scans with 4 digital scanners. J Prosthet Dent. 2020;123(1):85–95. 10.1016/j.prosdent.2019.02.008 30982616

[45] Osnes CA, Wu JH, Venezia P, Ferrari M, Keeling AJ. Full arch precision of six intraoral scanners in vitro. J Prosthodont Res. 2020;64(1):6–11. 10.1016/j.jpor.2019.05.005 31227447

[46] Park JM, Shim JS. Measuring the complete-arch distortion of an optical dental impression. J Vis Exp. 2019;(147). 10.3791/59261 31205295

[47] Song J, Kim M (2020). Accuracy on scanned images of full arch models with orthodontic brackets by various intraoral scanners in the presence of artificial saliva. Biomed Res Int.

[48] Jemt T, Hjalmarsson L (2012). In vitro measurements of precision of fit of implant-supported frameworks. A comparison between “virtual” and “physical” assessments of fit using two different techniques of measurements. Clin Implant Dent Relat Res.

